# Fracture triplane de l’extrémité supérieure du tibia: une lésion rare (étude d’un cas et revue de la littérature)

**DOI:** 10.11604/pamj.2019.32.46.9680

**Published:** 2019-01-28

**Authors:** Mohamed-Lemine Lehreitani, Hatim Abid, Florian Marcaillou, Maanouk Rachid, Abdelhalim El Ibrahimi, Abdelmajid El Mrini, Stephane Blanc

**Affiliations:** 1 Service de Chirurgie Orthopédique, Hôpital de Pontoise, Paris, France; 2 Service de Chirurgie Orthopédique et Traumatologie B4, CHU Hassan II Fès, Maroc

**Keywords:** Tibia proximale, fracture triplane, adolescent, ostéosynthèse, Proximal tibia, triplane fracture, adolescent, osteosynthesis

## Abstract

Nous rapportons le cas d’une fracture triplane de l’extrémité supérieure du tibia chez un jeune de 16 ans survenue dans les suites d’un accident de sport lors d’une course de vélo. La tomodensitométrie (TDM) a été réalisée pour une meilleure analyse lésionnelle. Après l’identification de tous les fragments nécessitant une ostéosynthèse, il a été réalisé à foyer fermé une réduction avec fixation des différents fragments fracturaires par des vis canulées. Au dernier recul, les résultats radiologiques et fonctionnels étaient excellents.

## Abstract (en)

We here report the case of a 16-year old patient with triplane fracture of the proximal end of the tibia due to a sporting accident occurred during a cycling race. CT scan (CT) was performed to assess the fracture with a high level of accuracy. After identification of all fragments requiring osteosynthesis, closed reduction was performed with fixation of fracture fragments using cannulated screws. At the last follow-up visit, radiological and functional results were excellent.

## Introduction

La fracture triplane de l’extrémité proximale du tibia est une lésion rare qui survient essentiellement chez l’adolescent. L'analyse de cette fracture basée sur des radiographies standards de face et de profil est souvent difficile. De ce fait, la tomodensitométrie (TDM) est fortement recommandée. Une réduction anatomique est particulièrement nécessaire pour diminuer le risque d’arthrose. La rééducation précoce est essentielle pour prévenir la raideur du genou.

## Patient et observation

**Figure 1:**
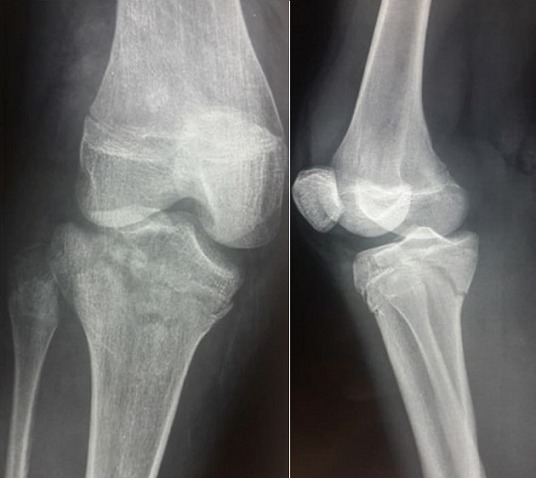
Radiographies standards de face et profil du genou droit révélant une fracture complexe de l’extrémité proximale du tibia

**Figure 2:**
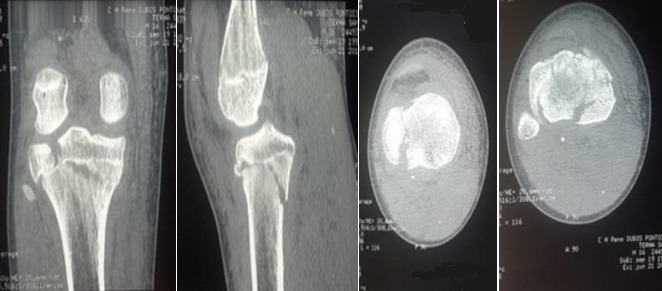
TDM préopératoire du genou droit confirmant le diagnostic d’une fracture triplane de l’extrémité proximale du tibia

**Figure 3:**
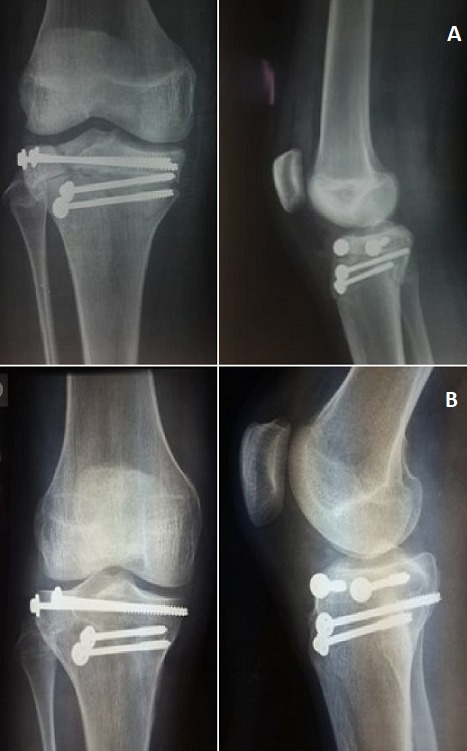
Radiographies post-opératoires: (A) post opératoire immédiat; (B) après un an de suivi

Un patient de 16 ans, admis pour la prise en charge d’un traumatisme fermé du genou droit. À l'examen, le patient était en bon état général. Le genou droit était tuméfié et douloureux à la palpation et la mobilisation, sans ouverture cutanée ni atteinte vasculo-nerveuse. La radiographie standard de face et de profil a révélé une fracture complexe de l’extrémité proximale du tibia (Figure 1). Le diagnostic d’une fracture triplane a été confirmé par la tomodensitométrie (Figure 2). A foyer fermé, nous avons procédé à une réduction et ostéosynthèse par des vis canulées (Figure 3). L’évaluation ligamentaire du pivot central et des ligaments collatéraux après l’ostéosynthèse n'a révélé aucune laxité. La rééducation passive du genou a été entamée dès le premier jour en post-opératoire. Les radiographies de contrôle à 8 semaines ont montré une consolidation complète de la fracture. Cliniquement, nous n’avons pas noté de limitation des amplitudes articulaires ni de laxité. Le patient a retrouvé ses activités quotidiennes et a repris le sport au bout de 3 mois.

## Discussion

Les fractures épiphysaires de l’extrémité proximale du tibia sont des lésions rares qui représentent 0,5 à 3% de toutes les fractures épiphysaires [1, 2]. Le siège et l’étendue du trait varient considérablement avec l'âge du patient. Les types I et II de la classification de Salter et Harris sont fréquents entre 10 et 12 ans. À l'adolescence, on observe une prédominance des fractures de type III et IV [3]. La soudure du cartilage de croissance de l’extrémité proximale du tibia se fait de façon asymétrique d'arrière en avant. Ceci favorise la survenue des fractures triplanes qui ont été décrites pour la première fois par Johnson et Fahl [4] en 1957.

Par définition, les fractures triplanes correspondent à une rupture de la continuité osseuse dans les 3 plans: sagittal, coronal et transversal. La localisation au niveau de l’extrémité distale du tibia est de loin la plus fréquente avec deux grandes séries de 86 et 51 cas rapportés respectivement par Jarvis *et al.* [5] et Brown *et al.* [6]. Cette entité lésionnelle a été décrite également sous forme de cas sporadiques au niveau des extrémités inférieures de l'humérus, du radius, du fémur ainsi qu’à la main [7-9]. Nous avons trouvé sept cas de fractures triplanes siégeant au tibia proximal [8-13]. Ainsi, nous rapportons dans ce travail le huitième cas de la littérature. Dans notre cas, la radiographie standard et la tomodensitométrie du genou ont objectivé une fracture triplane de l’extrémité proximale du tibia, comminutive, avec dans le plan frontal un fragment médial classé stade IV de Salter-Harris et un fragment latéral stade III. Dans le plan sagittal, on a noté un grand fragment classé stade II.

Les fractures épiphysaires du tibia proximal sont généralement des fractures qui surviennent dans les suites de traumatismes de haute énergie. En raison de la proximité des structures neurovasculaires et de l’atteinte de la plaque de croissance, ces fractures peuvent exposer les patients à des complications potentiellement graves. Le taux de complications varie de 5 pour cent pour l’atteinte de l'artère poplitée et le nerf fibulaire à 25 pour cent pour les troubles de croissance, les cals vicieuses et l’inégalité de longueur du membre [14, 15]. Dans un travail récent de Nowicki *et al.* [12], les auteurs rapportent la survenue d’un syndrome de loge de la jambe, chez un enfant de 11 ans qui présentaient une fracture triplane de l’extrémité proximale du tibia. Le risque de cette complication très grave est estimé à 3 pour cent [14]. Chez notre malade nous n’avons été confrontés à aucunes complications.

Sur le plan thérapeutique, tous les cas de fracture triplane du tibia proximal rapportés dans la littérature, ont bénéficié d'un traitement chirurgical. Conroy *et al.* [11] avait effectué, chez une fille de 11 ans, une réduction à foyer fermé pour corriger le déplacement postérieur dans le plan sagittal sous anesthésie générale avec genou en hyper-extension suivie d’une fixation en compression par une vis canulée 4,5mm par voie percutanée. Au dernier recul, les auteurs ne rapportent pas de perturbation de la croissance et la patiente a repris ses activités normales. Dans le cas publié par Nowicki *et al.* [12] intéressant une patiente de 11 ans, la réduction a été obtenue à ciel ouvert après échec de la tentative à foyer fermé en raison de l’incarcération des parties molles et du périoste dans le foyer fracturaire. Pour la fixation, les auteurs ont eu recours à des broches et des vis canulées. Les radiographies de contrôle après 12 mois, ont montré la consolidation de la fracture.

Cliniquement la patiente a retrouvé ses activités quotidiennes sans aucune limitation. Récemment, David WN *et al.* [13] ont publié un nouveau cas de fracture triplane du tibia proximal chez un adolescent de 14 ans survenue lors d’un match de football. La réduction s’est faite à ciel ouvert et la fixation a été réalisée à l'aide de vis canulées de 7mm de diamètre partiellement filetés. A 3 mois, le contrôle radiologique était satisfaisant et le patient a repris ses entraînements. Dans notre cas, nous avons opté pour une réduction à foyer fermée et une ostéosynthèse par des vis canulées 4,5mm. Le protocole de rééducation a été commencé un jour après la chirurgie. Après un an de suivi le score IKDC (International score Comité Knee Documentation) était de 100 sur 100. Au dernier recul, le patient pratiquait ses activités sportives avec le même niveau qu'avant la lésion, sans douleur, ni instabilité du genou et sans différence de force musculaire.

## Conclusion

Les fractures triplanes de l’extrémité proximale du tibia sont des lésions rares. Elles exigent une réduction anatomique de manière à prévenir l’évolution vers l'arthrose précoce. Malgré leur potentiel élevé de morbidité, en raison de l’atteinte du cartilage de croissance et de la proximité des structures neurovasculaires adjacentes à la face postérieure du genou, le traitement chirurgical bien conduit aboutit à un excellent résultat fonctionnel.

## Conflits d’intérêts

Les auteurs ne déclarent aucun conflit d'intérêts.
